# *Helicobacter pylori *infection is associated with decreased serum levels of high density lipoprotein, but not with the severity of coronary atherosclerosis

**DOI:** 10.1186/1476-511X-8-59

**Published:** 2009-12-23

**Authors:** En-Zhi Jia, Fu-Jun Zhao, Bo Hao, Tie-Bing Zhu, Lian-Sheng Wang, Bo Chen, Ke-Jiang Cao, Jun Huang, Wen-Zhu Ma, Zhi-Jian Yang, Guoxin Zhang

**Affiliations:** 1 Department of Cardiovascular Medicine, the First Affiliated Hospital of Nanjing Medical University, Nanjing 210029, Jiangsu Province, China; 2 Department of Gastroenterology, the First Affiliated Hospital of Nanjing Medical University, Nanjing 210029, Jiangsu Province, China

## Abstract

**Objective:**

The objective of this survey was to study the association between *Helicobacter pylori *infection and the severity of coronary atherosclerosis.

**Methods:**

The study population consisted of 961 consecutive patients (711 males and 250 females) who underwent coronary angiography for suspected or known coronary atherosclerosis. The patients' body mass index, blood pressure, the blood lipid, blood glucose, leukocyte count (10^9^/L), neutrophil count (10^9^/L), and Helicobacter *pylori*-specific IgG antibodies were performed. Coronary angiograms were scored according to vessel score and Gensini's score.

**Results:**

A significant association between *H. pylori *infection and coronary atherosclerosis as well as its severity was not find in this cross section study (*p *= 0.858). And, the level distribution of vessel score (*p *= 0.906) and Gensini's score (*p *= 0.905) were similar in the seropositivity group and seronegativity group of Helicobacter *pylori *infection. However, the level of fasting high-density lipoprotein cholesterol (mmol/L) (*p *= 0.013) was significantly lower in the seropositivity group than that in the seronegativity group of Helicobacter *pylori *infection.

**Conclusions:**

In conclusion, in the present study, a significantly correlation between Helicobacter *pylori *seropositivity and angiographically evaluated severity of atherosclerosis was not find. And, the present study showed a good correlation between Helicobacter *pylori *infection and decreased HDL cholesterol. However, the exact mechanisms need further study.

## Introduction

Cardiovascular diseases, including coronary atherosclerosis remain the leading cause of deaths in the developed and developing countries despite of declining mortality. Understanding the etiology and pathophysiology of coronary atherosclerosis is essential in treating the disease and prevent its subsequent consequences, such as myocardial infarction, and stroke. Coronary atherosclerosis is a multifactorial process where chronic inflammation plays a pivotal role, while other risk factors such as dyslipidemia also contribute to the pathogenesis of atherosclerosis [1,2]. It has been reported that the inflammation plays an important role in the initiation and progression of atherosclerosis and its complications [3]. A number of seroepidemiologic studies have suggested that there is an association between coronary atherosclerosis and several infectious agents, including those intracellular pathogens such as *Helicobacter pylori *and *Chlamydia pneumoniae *[4].

*H. pylori *is a bacterium that commonly colonizes the human stomach and causes chronic and active gastritis, peptic ulcer disease and is associated with increased risk of developing gastric cancer. Over the past decade, several studies have demonstrated that *H. pylori *infection is associated with the development of coronary atherosclerosis, and suggested a causal relationship although this issue is still controversial [5-10]. However, the mechanisms how *H. pylori *infection results in the coronary atherosclerosis, and the relationship between *H. pylori *infection and clinical and laboratory risk factors including blood pressure, smoking, blood glucose and lipids have not been fully understood. Moreover, it is unknown whether *H. pylori *infection is associated with the severity of coronary atherosclerosis. Therefore, the present cross-sectional study of 961 consecutive patients with angiographically confirmed coronary atherosclerosis was carried out to determine the association of *H. pylori *infection with any of the above clinical and laboratory risk factors and the severity of coronary atherosclerosis.

## Materials and methods

### Study subjects

From February 2004 to May 2006, consecutive adult patients with coronary atherosclerosis as confirmed by coronary angiography at the First Affiliated Hospital of Nanjing Medical University, Nanjing, China, were included in the study. Patients with spastic angina pectoris (i.e., acetylcholine-positive), infectious processes within 2 weeks prior to the catheterization, heart failure (Killip Class = 2) after acute myocardial infarction, hepatic dysfunction, vascular disease (aortitis treated with prednisolone), familial hypercholesterolemia, thyroid dysfunction, or adrenal dysfunction were excluded.

This study was approved by the Ethics Committee of the First Affiliated Hospital of Nanjing Medical University and informed consent was obtained from each patient.

### Coronary angiography

Coronary arteries were cannulated by the Judkins technique [11] with 5F catheters, and recorded on Kodak 35-mm cine film at a rate of 30 frames per second. When stenotic coronary arteries were found, a direct intracoronary injection of isosorbide dinitrate (2.5 mg/5 ml solution over 20 s) was performed, and the presence of stenosis was determined one minute after the injection by coronary angiography from several projections with help of a computer-assisted coronary angiography analysis system (Mipron 1; Kontron Co. Tokyo, Japan).

### Scoring of coronary angiogram

Coronary angiograms were scored according to vessel score and Gensini's score: Vessel score: This was the number of vessels with a significant stenosis (50% or greater reduction in lumen diameter). Scores ranged from 0 to 4, depending on the number of vessels involved. Left main artery stenosis was scored as single-vessel disease.

Gensini's score: The Gensini's score system is based on the hypothesis that the severity of coronary heart disease should be considered as a consequence of the functional significance of the vascular narrowing and the extent of the area perfused by the involved vessel or vessels. Based on the system, a Gensini score was computed by assigning a severity score to each coronary stenosis according to the degree of luminal narrowing and its geographic importance. Reduction in the lumen diameter, and the roentgenographic appearance of concentric lesions and eccentric plaques were evaluated (reductions of 25%, 50%, 75%, 90%, 99%, and complete occlusion were given Gensini scores of 1, 2, 4, 8, 16, and 32, respectively). Each principal vascular segment was assigned a multiplier in accordance with the functional significance of the myocardial area supplied by that segment: i.e. × 5 for the left main coronary artery, × 2.5 for the proximal segment of left anterior descending coronary artery (LAD), × 2.5 for the proximal segment of the circumflex artery, × 1.5 for the mid-segment of the LAD, × 1.0 for the right coronary artery, the distal segment of the LAD, the posterolateral artery, and the obtuse marginal artery, and × 0.5 for any others arteries [12].

### Anthropometric measurements

Anthropometric measurements were performed after the patients removed their shoes and upper garments and wore an examination gown. Height was measured to the nearest 0.1 cm using a wall-mounted stadiometer. Weight was measured to the nearest 0.1 kg using a hospital balance beam scale. Body mass index (BMI) was calculated as weight (kg) divided by the square of height (m^2^). Blood pressure was measured in the right arm with the participant seated and the arm bared, and three measurements were recorded for each individual, and the average was used as the reading.

### Detection of *H. pylori*-specific IgG antibodies

Serum samples were prepared from each of the participants. *H. pylori*-specific IgG antibodies were measured with the Assure(r) *H. pylori *Rapid Test with a current infection marker (CIM-test, Genelabs Diagnostics Pty Ltd, Singapore), which has been shown to have sensitivity, specificity, positive and negative predictive values, and accuracy of 93.2%, 90.5%, 94.9%, 87.5%, and 92.3%, respectively, in Chinese [13]. Briefly, CIM test was brought to room temperature from a 4°C freezer shortly before use. One drop of sample was placed on the test area. When the sample diffused across the membrane and touched the pink indicator line, Chase Buffer was added to the oval well and the tab marked "Hp" was pulled. The result was then interpreted in 15 minutes. The absence of the "A" band, which is a control line, indicated an invalid result. Two bands present both at the positions "B" and "C" (even faint) were suggestive of current infection and only one band present at the position "C" indicated previous exposure. All samples were tested by an investigator who was blinded to the results of the coronary angiography.

### Laboratory measurements

The total cholesterol (TCH), triglyceride (TG), fasting blood glucose (FBG), fasting high-density lipoprotein cholesterol (HDL-c), fasting low-density lipoprotein cholesterol (LDL-c) were determined by enzymatic procedures on an automated autoanalyzer (AU 2700 Olympus, 1st Chemical Ltd, Tokyo, Japan).

### Hematological measurements

Blood samples from every patient were drawn at admission to the coronary unit and the measurements including total leukocyte count (10^9^/L), neutrophil count (10^9^/L), were performed by the automated blood analyzer.

### Statistical analysis

Data analysis was performed by using the Statistical Package for Social Science (SPSS for Windows, version 10.0, 1999, SPSS Inc, Chicago, IL). Patients were classified into four groups according to the Gensini's scores (using the quartile values as cut-off points so that each group had an about equal number of patients to minimize any bias that may have been produced in the statistical analysis) and vessel score. In addition, patients were classified into two groups according to the status of *H. pylori *infection. Data of BMI was normally distributed and thus presented as mean ± standard deviation (SD) and comparisons were analyzed by the independent-sample T test and the one-way analysis of variance (ANOVA), whereas skewed data including age, systolic blood pressure (SBP, mmHg), diastolic blood pressure (DBP, mmHg), TG, FBG, TCH, HDL-c, LDL-c, leukocyte count (10^9^/L), neutrophil count (10^9^/L), vessel score and Gensini's scores were expressed as median and/or quartile ranges, and comparisons were analyzed by the Mann-Whitney U test or the Kruskal-Wallis test, where appropriate. Categorical variables including gender and *H. pylori *infection status were compared between the groups of patients by chi-squared analysis. Differences were considered to be statistically significant if the null hypothesis could be rejected with >95% confidence. All *P *values are 2-tailed.

## Results

### Demographical clincial and biochemical characteristics of study subjects

A total of 961 patients (711 males and 250 females, with a median (range) age of 63 (54~70 years) were enrolled in the study. The age, clinical and biochemical characteristics of these patients are shown in Table 1.

**Table 1:** Demographical and clinical and blood biochemical characteristics of the study subjects in relation to gender

Variables	Male (n = 711)	Female (n = 250)	Total (n = 961)	*T *or *Z*	*P*
Age (year)	63.00 (53.00-70.00)	64.00 (56.00-70.00)	63.00 (54.00-70.00)	-1.551	0.121
BMI(Kg/M^2^)	24.98 ± 3.01	24.76 ± 3.35	24.79 ± 3.39	0.788	0.431
SBP(mmHg)	130.00 (120.00-140.00)	138.00 (120.00-150.00)	130.00 (120.00-140.00)	-2.951	0.003
DBP(mmHg)	80.00 (70.00-85.00)	80.00 (70.00-85.00)	80.00 (70.00-85.00)	-0.672	0.502
Cholesterol (mmol/L)	3.96(3.40-4.47)	4.40(3.91-4.99)	4.07(3.49-4.60)	-7.300	0.000
Triglyceride (mmol/L)	1.37(0.98-2.00)	1.52(1.11-2.14)	1.41(1.00-2.02)	-1.905	0.057
Glucose (mmol/L)	4.75(4.31-5.48)	4.78(4.39-5.39)	4.77(4.34-5.48)	-0.914	0.361
HDL-c (mmol/L)	0.94(0.81-1.09)	1.12(0.97-1.29)	0.98(0.84-1.16)	-9.175	0.000
LDL-c (mmol/L)	2.29(1.87-2.76)	2.50(2.05-3.05)	2.36(1.90-2.84)	-4.286	0.000
Leukocyte count (10^9^/L),	6.50(5.30-8.30)	5.90(5.10-7.00)	6.30(5.20-8.00)	-4.398	0.000
Neutrophil count (10^9^/L)	3.99(3.03-5.48)	3.37(2.77-4.30)	3.80(2.97-5.12)	-5.505	0.000
HP (+/-)	440/271	156/94	596/365	0.021	0.885
vessel score	1.00 (0.00-3.00)	1.00 (0.00-2.00)	1.00 (0.00-2.00)	-5.103	0.000

Gensini's Score	25.00(4.00-64.00)	8.00(0.00-40.00)	20.00(2.00-58.00)	-5.318	0.000

Generally, SBP (*P *= 0.003), the levels of blood TCH (*P *< 0.001), HDL-c (*P *< 0.001), and LDL-c (*P *< 0.001) were significantly higher in females than those in males subjects. However, the level of leukocyte count (10^9^/L) (*P *< 0.001), neutrophil count (10^9^/L) (*P *< 0.001), vessel score (*P *< 0.001) and Gensini's score (*P *< 0.001) were significantly higher in males than those in female subjects. Of the patients, 596 patients (62.0%) were seropositive for *H. pylori *infection. The seropositivity rate was not significantly different between males (61.9%) and females (62.4%).

### Demographical, clinical and biochemical characteristics in patients according to Gensini's scores

Gensini scores ranged from 0 to 276.0, with a median (quartile ranges) of 20.0 (2.0-58.0), and vessel score ranged from 0 to 4, with a median (quartile ranges) of 1.00 (0.00-2.00). Table 2 and Table 3 show the demographical, clinical and biochemical characteristics in patients according to vessel score and Gensini's score. The frequency distribution of the gender differed significantly among the 4 groups (*P *< 0.001). Moreover, leukocyte count (10^9^/L) (*P *< 0.001), neutrophil count (10^9^/L) (*P *< 0.001), age (*P *< 0.001) and glucose (*P *< 0.001) were increased with the increase of vessel score and Gensini's score. And, HDL-c (*P *< 0.001) was decreased with the increase of vessel score and Gensini's score. However, BMI, SBP, DBP, TCH, triglyceride, and LDL-c did not change following the increase of the vessel score and Gensini's score (Table 2 and Table 3),

**Table 2:** Age and clinical and biochemical characteristics in patients grouped according to vessel score

Variables	Vessel score				*F *or *Chi-Square*	*P*
			
	0 (n = 324)	1 (n = 239)	2 (n = 175)	3 and 4 (n = 223)		
Age (year)	60.00 (52.00~67.00)	59.50 (50.00~69.00)	66.00 (57.00~72.00)	68.00 (62.00~72.00)	78.213	0.000
BMI (Kg/M^2^)	25.19 ± 3.20	24.79 ± 3.11	24.75 ± 2.92	24.75 ± 3.08	1.298	0.274
SBP (mmHg)	130.00(120.00~140.00)	130.00(120.00~140.00)	130.00(120.00~140.00)	130.00(120.00~150.00)	5.610	0.132
DBP (mmHg)	80.00(70.00~90.00)	80.00(70.00~85.00)	80.00(70.00~85.00)	80.00(70.00~80.00)	6.094	0.107
Cholesterol (mmol/L)	4.09(3.51~4.53)	4.01(3.36~4.66)	4.08(3.42~4.61)	4.09(3.63~4.65)	1.704	0.636
Triglyceride (mmol/L)	1.44(0.99~2.05)	1.41(0.98~2.09)	1.38(0.94~1.99)	1.40(1.10~1.96)	0.528	0.913
Glucose (mmol/L)	4.66(4.29~5.17)	4.74(4.29~5.41)	4.84(4.40~5.61)	5.00(4.44~6.18)	23.548	0.000
HDL-c (mmol/L)	1.04(0.90~1.21)	0.99(0.82~1.11)	0.95(0.81~1.15)	0.94(0.81~1.11)	22.088	0.000
LDL-c (mmol/L)	2.31(1.92~2.76)	2.32(1.83~2.85)	2.31(1.81~2.99)	2.50(2.02~2.93)	6.451	0.092
Leukocyte count (10^9^/L),	5.7(4.85-6.80)	6.30(5.20-8.20)	6.40(5.50-8.50)	7.00(5.70-9.00)	63.422	0.000
Neutrophil count (10^9^/L)	3.30(2.60-4.20)	3.90(3.10-5.30)	4.10(3.06-5.48)	4.34(3.30-6.23)	69.224	0.000
Gender (M/F)	209/115	180/59	135/40	185/38	26.801	0.000
*H. pylori *(+/-)	125/199	86/153	66/109	88/135	0.648	0.885

**Table 3:** Age and clinical and biochemical characteristics in patients grouped according to Gensini's score

Variables	Gensini's score				*F *or *Chi-Square*	*P*
			
	~2.00 (n = 247)	2.01~20.00 (n = 234)	20.01~58.00 (n = 238)	58.01~(n = 242)		
Age (year)	58.00 (50.00~65.00)	63.00 (54.00~70.50)	64.00 (55.00~71.00)	66.00 (58.00~72.00)	54.883	0.000
BMI (Kg/M^2^)	25.30 ± 3.29	24.75 ± 2.92	24.74 ± 3.04	24.82 ± 3.11	1.811	0.144
SBP (mmHg)	130.00(120.00~140.00)	130.00(120.00~145.00)	130.00(120.00~140.00)	130.00(120.00~145.00)	4.494	0.213
DBP (mmHg)	80.00(70.00~90.00)	80.00(70.00~85.00)	80.00(70.00~85.00)	80.00(70.00~85.00)	5.152	0.161
Cholesterol (mmol/L)	4.04(3.51~4.46)	4.13(3.56~4.67)	4.09(3.44~4.69)	4.02(3.53~4.57)	2.810	0.422
Triglyceride (mmol/L)	1.44(0.99~2.00)	1.38(0.95~2.08)	1.45(1.08~2.07)	1.35(1.02~1.97)	1.767	0.622
Glucose (mmol/L)	4.66(4.29~5.17)	4.71(4.32~5.26)	4.84(4.27~5.54)	5.06(4.47~6.22)	28.978	0.000
HDL-c (mmol/L)	1.04(0.90~1.23)	1.03(0.87~1.17)	0.92(0.80~1.06)	0.96(0.82~1.12)	34.730	0.000
LDL-c (mmol/L)	2.29(1.89~2.72)	2.39(1.89~2.86)	2.32(1.87~2.98)	2.47(1.95~2.93)	3.193	0.363
Leukocyte count (10^9^/L)	5.60(4.80-6.70)	6.05(5.10-7.38)	6.905.50(-8.50)	7.20(5.70-9.45)	78.189	0.000
Neutrophil count (10^9^/L)	3.20(2.60-4.20)	3.52(2.60-4.50)	4.11(3.20-5.40)	4.60(3.29-6.83)	86.715	0.000
Gender (M/F)	157/90	166/68	188/50	198/44	26.359	0.000
*H. pylori *(+/-)	92/155	92/142	87/151	96/146	0.763	0.858

### Associations of *H. pylori *infection with demographical, clinical and biochemical variables and the severity of coronary atherosclerosis in patients with coronary atherosclerosis

Table 4 shows the associations of *H. pylori *infection with clinical and biochemical variables and the severity of coronary atherosclerosis in patients. There was no difference in the gender distribution and the age between *H. pylori *positive and negative patients. BMI, SBP, DBP, TCH, triglyceride, blood glucose, LDL-c, leukocyte count (10^9^/L), neutrophil count (10^9^/L) were also similar between the two groups. However, the level of HDL-c was significantly lower in the seropositive patients than in the seronegativity group (0.97 mmol/L vs.1.02 mmol/L, *P *= 0.013) (Table 3). There was no significant deference in the positive rate among patients with different vessel score and Gensini's score; in other words, there was no significant association between *H. pylori *infection and the severity coronary atherosclerosis (Tables 2, 3, 4).

**Table 4:** Age and clinical and biochemical characteristics in patients grouped according to the status of helicobacter pylori infection

Variables	Helicobacter pylori negative (n = 365)	Helicobacter pylori positive(n = 596)	*T *or *Z*	*P*
Age (year)	64.00 (55.00-71.00)	62.50 (53.25-70.00)	-1.245	0.213
BMI (Kg/M^2^)	25.15 ± 3.03	24.79 ± 3.15	1.697	0.090
SBP (mmHg)	130.00 (120.00-140.00)	13000 (120.00-145.00)	-0.265	0.791
DBP (mmHg)	80.00 (70.00-85.00)	80.00 (70.00-85.00)	-1.384	0.166
Cholesterol (mmol/L)	4.10(3.51-4.61)	4.05(3.49-4.58)	-0.504	0.614
Triglyceride (mmol/L)	1.40(0.98-2.01)	1.42(1.01-2.06)	-0.370	0.711
Glucose (mmol/L)	4.77(4.32-5.56)	4.77(4.37-5.43)	-0.011	0.991
HDL-c (mmol/L)	1.02(0.86-1.19)	0.97(0.83-1.13)	-2.494	0.013
LDL-c (mmol/L)	2.32(1.87-2.84)	2.37(1.93-2.84)	-1.148	0.251
Leukocyte count (10^9^/L)	6.20(5.18-7.93)	6.30(5.30-8.00)	-0.812	0.417
Neutrophil count (10^9^/L)	3.70(2.90-5.10)	3.83(3.00-5.20)	-0.870	0.384
Gender(M/F)	271/94	440/156	0.021	0.885
vessel score	1.00(0.00-2.00)	1.00(0.00-2.00)	-0.118	0.906

Gensini's Score	20.00(2.00-60.00)	20.50(2.00-57.00)	-0.120	0.905

## Discussion

The role of inflammation mechanism in the pathogenesis and progression of coronary artery disease has been increasingly explored, but still remains to be elucidated. Epidemiological studies based on serological findings have suggested an association between chronic *H. pylori *infection and atherosclerosis, although controversies exist [8,14,15].

A few studies explored the association between *H. pylori *infection and the clinical and biochemical variables in patients with atherosclerosis. The present study showed that HDL-c was significantly decreased in patients with *H. pylori *infection, indicating that *H. pylori *infection may resulted in decreased blood HDL-c levels, which then contributes to the development of coronary atherosclerosis. *H. pylori *infection has been suggested to influence the development of atherosclerotic changes in coronary arteries, indicating a damaging effect of this bacterium or its products (e.g. cytokines, endotoxins, cytotoxins and other virulence factors) on the coronary endothelium [16]. In a study of 470 healthy blood donors and 238 patients with angiographically proven coronary heart disease, the mean HDL-c concentration was significantly decreased in *H. pylori *-positive healthy subjects compared with *H. pylori *-negative subjects (1.36 vs. 1.44 mmol/L, *P *= 0.006) in unadjusted analysis. In addition, the association between *H. pylori *infection and decreased HDL-c persisted and remained significant in multivariate linear regression analysis (*P *= 0.002) [17]. This finding is further supported by our present study. Therefore, it seems that chronic *H. pylori *infection results in decreased HDL-c levels, and these lipid alterations could, at least in part, contribute to the initiation and development of coronary atherosclerotic diseases in *H. pylori *infected individuals. However, the underlying mechanisms and potential pathogenic pathways remained to be revealed.

In the present study, the seropositive rate for *H. pylori *infection in the Chinese patients with coronary atherosclerosis is of 62.0%. This rate is close to the prevalence of *H. pylori *infection in Chinese patients with dyspeptic symptoms (65% reported in 1991 by Li et al [18], and 61% reported in 1995 by Zhou and Yang [19]), and in individuals with a high gastric cancer risk (62% reported in 2008 by Shi et al [20]) but higher than that reported in healthy volunteers (49% reported in 1991 by Li et al [18], and 43% reported in 1995 by Zhou and Yang [19]). In 2007, Chen et al reported that the age-specific seroprevalence of *H. pylori *infection had been decreased from 56% in 1993 to 47% in 2003 in healthy Chinese population in Guangzhou city [21]. It is conceivable that the seropositive rate (62%) observed in patients with coronary atherosclerosis is higher than that in Chinese healthy population, implying that *H. pylori infection *in patients with coronary atherosclerosis is associated with the initiation and development of coronary atherosclerosis. However, a healthy control group was not included in the present study since the aim of the study was to determine association of *H. pylori *infection with major biochemical variables and severity of coronary atherosclerosis. Nevertheless, most previous epidemiological studies demonstrated an association between *H. pylori *infection and coronary atherosclerosis, suggesting a causal relation. Therefore, we postulated that, in addition to the possible role in the initiation of coronary atherosclerosis, chronic *H. pylori *infection would accelerate the severity of the disease. However, another studies demonstrated that *H. pylori *infection may not be an important factor in determining the risk of coronary artery disease [15,8], there is no data on the association between *H. pylori *infection and the severity of coronary atherosclerosis. In the present study, a significantly association between *H. pylori *seropositivity and angiographically evaluated severity of coronary atherosclerosis was not revealed.

A few mechanisms may explain this finding. First, chronic *H. pylori *infection may not play a role in the progression of coronary atherosclerosis once the disease has been induced. Second, the Gensini scores indicate the severity degree of stenotic coronary lesion which occurs especially in the late phase of coronary atherosclerosis, while the inflammation following *H. pylori *infection contributes to the early stage of atherosclerosis [22]. Therefore, indicators other than Gensini scores may be also used in the future studies on the association between *H. pylori *infection and the severity of coronary atherosclerosis. In addition, it has been reported in a meta-analysis that *cagA *positive *H. pylori *strains that are more virulent than *cagA *negative strains, are associated with an increased risk of developing atherosclerotic diseases including both ischemic stroke and coronary heart disease [23] However, in the present study, *cagA *status was not determined, and thus it is unknown whether *cagA *positive strains are associated with the severity of the coronary atherosclerosis. As described above, there are some limitations in the present study. First, the present study is only a cross-sectional study rather than a prospective, case-control study, and thus the obtained findings cannot provide information regarding the cause and effect relationship between *H. pylori *infection and coronary atherosclerosis. Second, virulence factors of *H. pylori *such as *cagA *gene and *H. pylori*-induced immune/inflammatory factors were not detected in the present study, which does not allow further speculation of the mechanisms how *H. pylori *infection may contribute to the initiation and development of coronary atherosclerosis. In conclusion, there is a significantly association between *H. pylori *seropositivity and decreased blood HDL-c levels. However, an association between H. pylori seropositivity and angiographically evaluated severity of coronary atherosclerosis was not found.

## Competing interests

The authors declare that they have no competing interests.

## Authors' contributions

EZJ participated in the design of the study and performed the statistical analysis.

FJZ carried out the immunoassays.

LSW, BC, KJC, JH, WZM, ZJY conceived of the study, and participated in its design and coordination.

TBZ and GZ participated in the design of the study

All authors read and approved the final manuscript.
